# Variations of Brain Functional Connectivity in Alcohol-Preferring and Non-Preferring Rats with Consecutive Alcohol Training or Acute Alcohol Administration

**DOI:** 10.3390/brainsci11111474

**Published:** 2021-11-07

**Authors:** Yue Liu, Binbin Nie, Taotao Liu, Ning Zheng, Zeyuan Liu, Baoci Shan, Lihong Jiang, Anne Manyande, Xihai Li, Fuqiang Xu, Jie Wang

**Affiliations:** 1 Key Laboratory of Magnetic Resonance in Biological Systems, State Key Laboratory of Magnetic Resonance and Atomic and Molecular Physics, National Center for Magnetic Resonance in Wuhan, Wuhan Institute of Physics and Mathematics, Innovation Academy for Precision Measurement Science and Technology, Chinese Academy of Sciences—Wuhan National Laboratory for Optoelectronics, Wuhan 430071, China; liuyue@wipm.ac.cn (Y.L.); liutaotao1101@163.com (T.L.); zhengning1993@163.com (N.Z.); zyliusky@whu.edu.cn (Z.L.); 2 Key Laboratory of Nuclear Radiation and Nuclear Energy Technology, Institute of High Energy Physics, Chinese Academy of Sciences, Beijing 100049, China; niebb@ihep.ac.cn (B.N.); shanbc@ihep.ac.cn (B.S.); 3 University of Chinese Academy of Sciences, Beijing 100049, China; 4 Magnetic Resonance Research Center, Yale University, New Haven, CT 06511, USA; lihong.jiang@yale.edu; 5 School of Human and Social Sciences, University of West London, Middlesex TW8 9GA, UK; Anne.Manyande2@uwl.ac.uk; 6 Academy of Integrative Medicine, Fujian University of Traditional Chinese Medicine, Fuzhou 350122, China; lixihaifz@163.com; 7 Center for Excellence in Brain Science and Intelligence Technology, Chinese Academy of Sciences, Shanghai 200031, China; 8 Wuhan National Laboratory for Optoelectronics, Huazhong University of Science and Technology, Wuhan 430074, China

**Keywords:** alcohol-preferring rats, fMRI, genetic background, functional connectivity, resting state

## Abstract

Alcohol addiction is regarded as a series of dynamic changes to neural circuitries. A comparison of the global network during different stages of alcohol addiction could provide an efficient way to understand the neurobiological basis of addiction. Two animal models (P-rats screened from an alcohol preference family, and NP-rats screened from an alcohol non-preference family) were trained for alcohol preference with a two-bottle free choice method for 4 weeks. To examine the changes in the neural response to alcohol during the development of alcohol preference and acute stimulation, different trials were studied with resting-state fMRI methods during different periods of alcohol preference. The correlation coefficients of 28 regions in the whole brain were calculated, and the results were compared for alcohol preference related to the genetic background/training association. The variety of coherence patterns was highly related to the state and development of alcohol preference. We observed significant special brain connectivity changes during alcohol preference in P-rats. The comparison between the P- and NP-rats highlighted the role of genetic background in alcohol preference. The results of this study support the alterations of the neural network connection during the formation of alcohol preference and confirm that alcohol preference is highly related to the genetic background. This study could provide an effective approach for understanding the neurobiological basis of alcohol addiction.

## 1. Introduction

As one of the most pervasive mental disorders or endogenous depression, alcoholism affects approximately 10% of Americans at some time in their lives [1]. First, drinking alcohol not only changes the gut environment, but also modulates the composition of gut microbiota and is associated with alcohol-related diseases [2]. Second, high alcohol intake or withdrawal is particularly detrimental to cerebral function and may lead to brain impairments [3]. Furthermore, alcoholism is always correlated with secondary effects, such as irresponsible or asocial behavior and incidences of suicide, violence, or impaired driving. Thus, investigations of the reasons for alcohol abuse are very important for solving social problems.

The major risk factors for alcohol abuse or dependence are related to family history (genetic reasons) or other common bad habits/environmental factors [4]. Alcohol drinking preference or abuse in both experimental animals and humans is influenced by genetic factors, which means the offspring of alcoholic parents have a higher risk of becoming alcohol abuse patients than those of non-alcoholic parents [5]. Furthermore, environmental factors, such as stress, and bad drinking habits are also associated with heavy drinking [6,7]. However, the contribution of these two factors has been almost contradictory in previous investigations [8,9]. Regardless of the sources of alcohol abuse, a binge alcohol drinking pattern exhibits anomalous neural activity, which may reflect underlying dysfunctions in neurophysiological mechanisms [10].

Although there are differences between functional and structural connectivity in alcohol abuse subjects [11], it is very difficult to follow up with the changes of the whole brain connectivity during the process of alcohol preference or addiction. As an important noninvasive tool for further understanding the brain functions during different diseased states, functional magnetic resonance imaging (fMRI) was first utilized in humans [12] and then in nonhuman primates [13]. Using fMRI, it was found that alcohol abuse is always associated with iron accumulation in deep grey matter [14] or deficits of the regional cerebral blood flow (CBF) [15]. Human fMRI studies require long-term (several years) follow-up of the volunteers [16] or pure family history background [17]. Thus, animal models have become more widespread in the study of various human diseases and genetic traits. However, there is limited research on the variations in functional connectivity in the whole brain related to alcohol abuse due to the limited brain templates for rats. Since the first version of the brain template was reported [18,19], only a few whole-brain resting-state functional magnetic resonance imaging (rsfMRI) results have been published using animal models [20,21]. To our knowledge, no common rat brain template has been published with comparisons to human studies. Functional connectivity is defined as the temporal correlation of low-frequency (0.01–0.1 Hz) fluctuations of the BOLD signal between spatially distinct brain regions [22]. As a noninvasive examination or localization technology, rsfMRI is a valuable tool for investigating the network level of the brain under healthy and diseased states. A rat brain template [19] was used to analyze the functional network of the retrosplenial cortex in the whole brain. Using a similar strategy, the rsfMRI method was utilized to investigate the whole-brain resting state under different conditions considering the family history and different alcohol training periods.

To verify the effect of alcohol training and family history on the functional network of the brain, alcohol-preferring rats (P-rats) and alcohol-non-preferring rats (NP-rats) were utilized for rsfMRI analysis. The method of two-bottle free choice was used to train the animals for alcohol preference screening, and the whole-brain correlation coefficients of three different training periods (Pre-, Mid-, Post-) during the alcohol preference formation with P- and NP-rats were analyzed.

## 2. Methods and Materials

### 2.1. Animals

The entire experiment was performed according to the Regulations of the Chinese Council on Animal Care, approved by the Animal Care and Use Committee of Wuhan Institute of Physics and Mathematics, Chinese Academy of Science, and reported following the ARRIVE guidelines. The breeding pairs of alcohol-preferring and non-preferring rats (P-rats and NP-rats, respectively) were provided as gifts from the University of Melbourne (Prof. Andrew John Lawrence) with permission from the School of Medicine, Indiana University. All the animals were bred in-house and acclimated to single housing, kept under a standard 12 h light–dark cycle, and given food ad libitum at an optimal environmental temperature (23~25 °C). Male P-rats (n = 12; Pre-: n = 12, Mid-: n = 9, Post-: n = 8 for fMRI analysis; two rats died during the fMRI data acquisition in the Mid- period, and three imaging data (Mid-: n = 1; Post-: n = 2) were discarded due to uncorrectable motion and noise) and male NP-rats (n = 9) (200–250 g at the time of training) were used for fMRI experiments. 

### 2.2. Alcohol Preference Training

Single-housed P- and NP-rats (male, ~200 g) had continuous access to one bottle of 5% alcohol (*v*/*v*) and one bottle of water in their home cage for four consecutive weeks according to a two-bottle free choice (2-BC) paradigm [23,24]. The mass of drinking liquid (5% alcohol and water) was monitored every day, and the bottles were randomly replaced every day. If the alcohol consumption ratio was higher than 80% (P-rats) for three consecutive days, then that animal was treated as successfully trained for alcohol preference. However, if this ratio was lower than 20% (NP-rats), then the animal was treated as alcohol-non-preferring.

### 2.3. Resting-State fMRI Experiments

At first, the whole-brain level MRI scans of P- and NP-rats were designed in three different periods (P-Pre: before alcohol training, P-Mid: 2 weeks after alcohol training; P-Post: 4 weeks after alcohol training) and two different states (RS: resting state; IS: intoxicated state/acute alcohol treatment). The MRI experiments were conducted using a Bruker Biospec 70/20 USR small animal MR system (Bruker BioSpin MRI, Ettlingen, Germany) operating at 7.0 T. The complete steps for the experimental procedure were as follows.

All the animals were initially anesthetized with isoflurane (3.0–5.0% for induction and set-up on the animal bed). They were kept in an MRI scanner breathing isoflurane (0.6–1.2% for light anesthesia) in oxygen-enriched air (20% O_2_ and air). Breathing rates, heart rates, and blood oxygen saturation levels were monitored using a pulse oximeter positioned at the hind limb and a pressure-sensitive sensor under the abdomen (MR-compatible Small Animal Monitoring & Gating System, SA Instruments, Inc., New York, NY, USA). The body temperature of each rat was maintained at ~37 °C using a warm water circuitry system. A needle connected to a PE-50 tube was implanted in the abdomen for the administration of the alcohol solution (0.76 g/kg i.p.) from outside the magnet. A planar receive coil surface coil with a diameter of 20 mm was placed on top of the skull and utilized in combination with a detunable partial volume transmit coil (Bruker BioSpin MRI, Ettlingen, Germany).

A T2 anatomical reference scan in the coronal plane was acquired using a spin-echo (Turbo-RARE) sequence: field of view (FOV) = 25.6 × 25.6 mm^2^; matrix dimension (MD) = 192 × 192; repetition time (TR) = 3000 ms; echo time (TEeff) = 33 ms; RARE factor = 8; number of averages (NA) = 9; spatial resolution = 0.13 × 0.13 × 0.8 mm^3^; 16 slices without gaps. The resting-state (rsfMRI or RS) datasets were then acquired using single-shot gradient-echo EPI (Echo Planar Imaging) with the following parameters: FOV: 25.6 × 25.6 mm^2^; MD: 64 × 64; TR: 2000 ms; TE: 16 ms; 16 different coronal slices of the same locations with a T2 anatomical image; voxel dimensions: 0.4 × 0.4 × 0.8 mm^3^, bandwidth: 200 kHz (6250 Hz/voxel). Each resting-state fMRI scan comprised 320 repetitions, and the duration of each scan was around 10 min 40 s. After the resting state analysis, the animal was treated with acute ethanol administration.

The experiment of the intoxicated state (IS) was defined as the period of 15 min~1 h after the alcohol injection (0.76 g/kg i.p.). The functional scans (RS and IS) were acquired per rat when the subject was under light anesthesia and immobility (estimated by the respiratory rate stabilized at 80–100 per minute). 

### 2.4. Resting-State fMRI Analysis

#### Data Analysis

The post-processing of all the functional images was performed by a single experienced observer who was unaware of to whom the scans belonged. The preprocessing and data analysis were performed using a homemade toolbox with MATLAB code (spmratIHEP) [25] in the statistical parametric mapping (SPM8) software (http://www.fil.ion.ucl.ac.uk/spm (accessed on 8 April 2020), which comprised an fMRI rat brain template [19] and an atlas in the Paxinos and Watson space.

To calculate the whole-brain network of rsfMRI, the files of Bruker MR image data were first converted to the imaging analyzing format with the Micro Bruker2Anlyzer Converter (Bru2Anz) software. Then, the functional datasets of all the individuals were preprocessed in spmratIHEP using the following steps:

(1) *Data screening*: The first 10 volumes of each scan were discarded to allow for magnetization equilibrium.
(2) *Slice timing*: The differences in slice acquisition times of each scan were corrected using the slice timing approach.
(3) *Realignment*: The temporal processed volumes of each subject were realigned to the first volume to remove the head motion, and a mean image was created over the 310 realigned volumes. All the participants had less than 1 mm of translation in the x-, y-, or z-axis and 1° of rotation in each axis.
(4) *Spatial normalization*: The realigned volumes were spatially standardized into the Paxinos and Watson space by normalizing with the EPI template of rat brain via their corresponding mean image. Then, all the normalized images were resliced by 1.0 × 1.5 × 1.0 mm^3^ voxels.
(5) *Smooth*: The normalized functional series were smoothed with a Gaussian kernel of 2 × 4 × 2 mm^3^ FWHM (full width at half-maximum).

Using DPARSF (http://rfmri.org/DPARSF (accessed on 8 June 2020), all smoothed images were then bandpass-filtered at 0.01–0.08 Hz and further corrected for the effect of head movement by regressing the translations and rotations of the head estimated during image realignment. 

**Figure 1:**
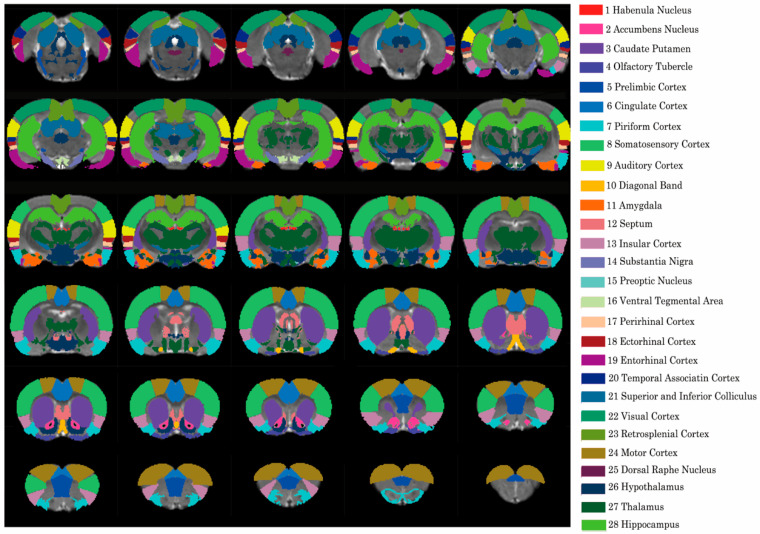
Illustration of the 28 selected brain regions in the structure of the rat brain.

Considering the resolution of the fMRI data and brain regions related with alcohol preferences, the whole rat brain was automatically divided into 28 different regions of interest (ROIs) across both hemispheres based on physiological structures and functions using the brain template [19]. The functional connectivity was evaluated using seed-based correlational analyses with a similar former method [26] (Figure 1). The time courses from all the voxels within the individual seed regions were averaged and used as reference time courses. Pearson’s cross-correlation coefficients among the reference time courses, as well as the time course of each voxel, were then calculated and used to quantify the strength of the functional connectivity. Finally, for each rat, a functional connectivity matrix was established.

### 2.5. Statistical Analysis

The statistical analyses of the functional connectivity data of all the animals in different groups (different states: RS and IS; different periods: P-Pre, P-Mid, and P-Post) were performed with MATLAB (MathWorks, Inc., Natick, MA, USA). Pearson’s cross-correlation coefficients were used to analyze the difference of the whole-brain networks among the different groups. Student’s *t*-test and ANOVA with a post-hoc test (LSD) were used to compare the two states and three periods. With equal variance not assumed, a nonparametric test (Dunnett T3) was used to compare the differences between the groups. The *p*-values were adjusted for multiple testing with the “fdr” or its alias “BH” method. The results in the text are displayed as the means ± SD. In general, *p*-value of 0.05 was considered statistically significant, * *p* < 0.05; ** *p* < 0.01; **** *p* < 0.001.

## 3. Results

### 3.1. Detection of Alcohol Preference Using Two-Bottle Free Choice

**Figure 2:**
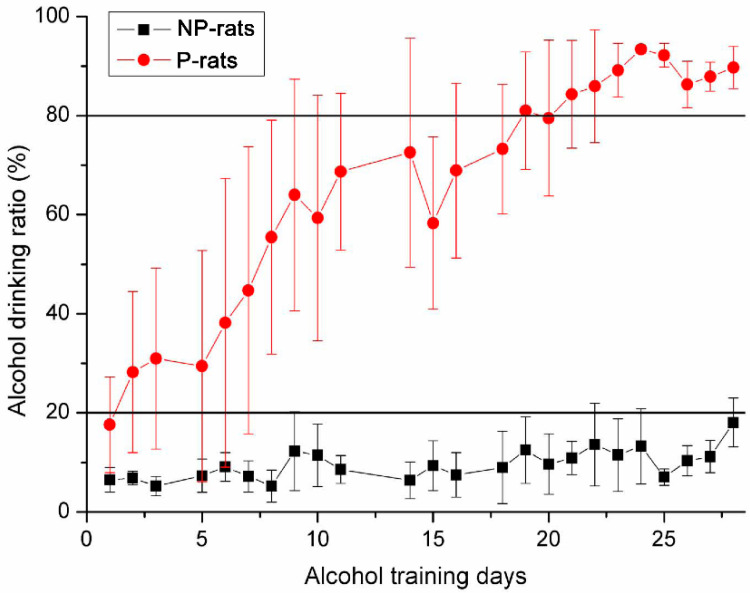
Results of the two-bottle free choice of the alcohol drinking training for the alcohol-preferring rats (P-rats) and the alcohol-non-preferring rats (NP-rats).

With training using the two-bottle free choice method, the P- and NP-rats showed significantly different patterns of alcohol preference drinking (Figure 2). For the NP-rats, the alcohol drinking ratio was <20% throughout the whole 4-week training period, which was mostly caused by leaks from the bottles or limited alcohol drinking. However, for the P-rats, the alcohol drinking ratio gradually increased following the training periods and ultimately reached >80%, which was regarded as the development of alcohol preference behavior. According to the drinking rate curve, three different periods were defined from primary alcohol drinking to alcohol preference: P-Pre, P-Mid (2-week alcohol training), and P-Post (4-week alcohol training).

### 3.2. Whole-Brain Network Analysis of the Resting State

**Figure 3:**
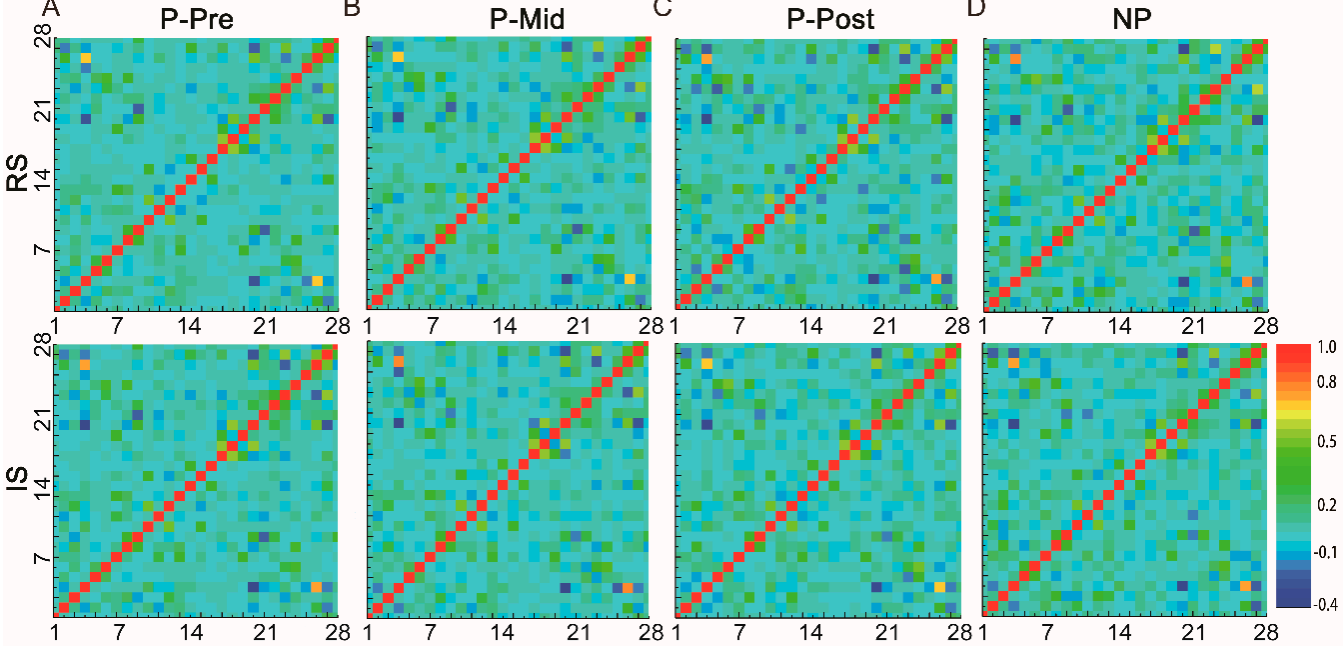
The mean functional connectivity matrices of the P-rats between different periods (alcohol drinking training period: week 0, 2 weeks, and 4 weeks; alcohol stimulation) and the NP-rats. Note, upper row: resting state analysis; lower row: acute alcohol stimulation; (**A**): P-Pre, week 0; (**B**): P-Mid, 2 weeks; (**C**): P-Post, 4 weeks; (**D**): NP. The horizontal and vertical coordinates are brain region numbers in Figure 1.

To investigate the relationship of the brain network and different periods of alcohol preference (P-Pre, P-Mid, and P-Post) / the effects of alcohol stimulation (RS and IS), a series of EPI scans under different treatment periods and alcohol acute stimulation was performed (Figure 3). 

The Pearson’s cross-correlation coefficients across these different brain-wide ROIs were calculated, and the mean functional connectivity matrices of the P- and NP-rats between different periods and stages are illustrated in Figure 3A–D. From the global perspective, the patterns of the whole-brain connection showed massive agreements between different treatment periods and different stimulation states. The correlation coefficients of the functional connectivity ranged from ~0.84 to 0.96 (Table 1).

**Figure 4:**
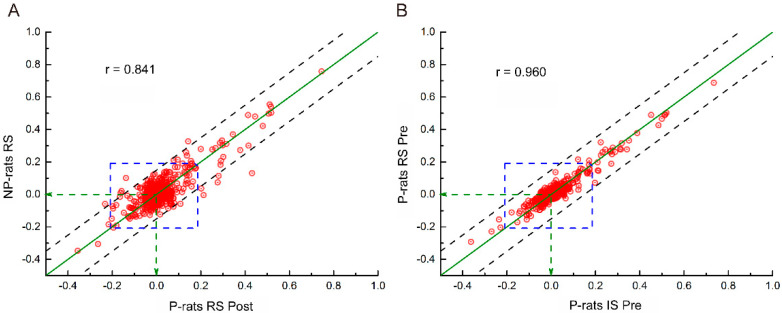
Examples of the correlation of the resting state functional connectivity in the whole brain for the P- and NP-rats. (**A**): P-rats RS Post vs. NP-rats RS; (**B**): P-rats IS Pre vs. P-rats RS Pre; Note: point, functional connectivity of two brain regions; dotted lines, y = x ± 0.15; area between the two dotted lines, the difference of the two examples was smaller than 0.15.

To clearly show the effects of alcohol preference, two different kinds of animals (P-rats and NP-rats) under the state of RS were compared (R = 0.841, *p* < 0.005, Figure 4A), and another example from P-rats under different states (RS and IS) in the P-Pre group was also provided for comparison (R = 0.960, *p* < 0.005, Figure 4B). Among these coefficients, the different periods of alcohol preference and alcohol stimulation ranged from 0.910 to 0.960, which is much higher than the comparison between the NP-rats and these groups (from 0.841 to 0.878, *p* < 0.005).

**Figure 5:**
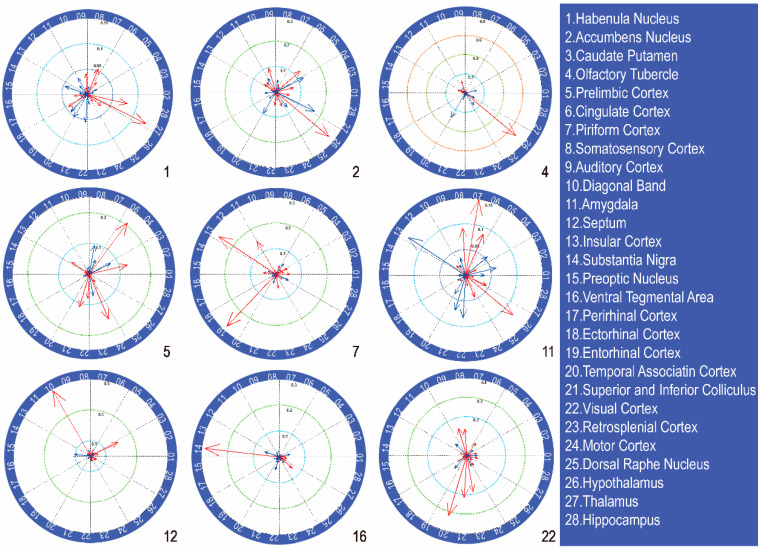
Examples of functional connectivity for nine different selected brain ROIs. The red arrows are positive correlations, and the blue ones are negative. The arrow length is proportional to the numerical value. Note: 1—Habenula nucleus; 2—Accumbens nucleus; 4—Olfactory tubercle; 5—Prelimbic cortex; 7—Piriform cortex; 11—Amygdala; 12—Septum; 16—Ventral tegmental area; 22—Visual cortex.

To demonstrate the reliability of the coefficients and compare the connectivity of the different brain regions clearly, the connectivity properties of several alcohol-preference-associated brain regions (habenula nucleus; accumbens nucleus; olfactory tubercle; prelimbic cortex; piriform cortex; amygdala; septum; ventral tegmental area; visual cortex) with a well-known projection relationship were extracted and are shown in Figure 5. For instance, relatively high correlation coefficients were found in the accumbens nucleus and hypothalamus (2), piriform cortex and entorhinal cortex (7), and ventral tegmental area and substantia nigra (16).

### 3.3. Variations of Functional Connectivity with Alcohol Stimulation and Alcohol Preference

To investigate the relationship between brain connectivity and alcohol stimulation or alcohol preference, the whole-brain connectivity of different brain regions (756 pairs in each state and period) under different states (periods and stimulation conditions) was compared.

**Figure 6:**
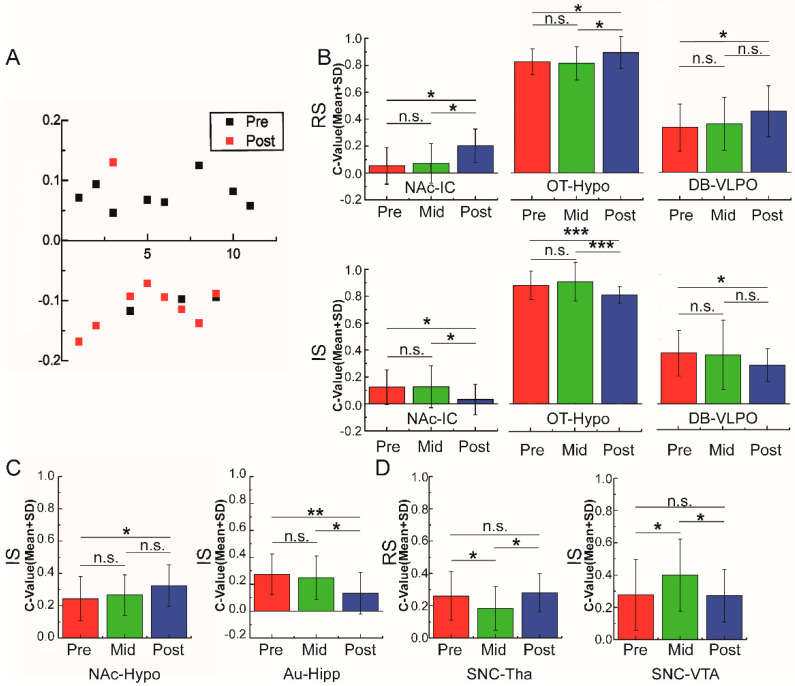
Different patterns of change among the analyses of functional connectivity for the whole brain. (**A**) Tendency of the changes for the acute alcohol stimulation during the Pre- and Post- preference periods, Δ*R* = IS(*r*) − RS(*r*). (**B**) The functional connectivity increased during the resting state but decreased after acute alcohol stimulation after alcohol preference. (**C**) The changes of the functional connectivity only happened during the alcohol preference formation period. (**D**) The functional connectivity only changed during the period of alcohol acute stimulation. The abbreviations of brain areas are the same as in the main text: NAc (nucleus of accumbens), OT (olfactory tubercle), VTA (ventral tegmental area), IC (insular cortex), Hypo (hypothalamus), SNC (compacta of substantia nigra), Au (auditory cortex), DB (diagonal band), Hipp (hippocampus), VLPO (ventrolateral preoptic nucleus); * *p* < 0.05, ** *p* < 0.01, *** *p* < 0.005.

At first, the changes of the brain functional connections under ethanol stimulation in different periods for P-rats (pre-preference and post-preference) were calculated and compared. With the stimulation of ethanol injection, the connectivity of 11 paired regions of the pre-preference periods and nine paired regions of the post-preference period had significant changes (*p* < 0.05, Table S1). The tendency of the changes in the brain connections of these regions was collected (Figure 6A). There was a clear opposite tendency between different preference periods: mostly increasing (8/11) in the pre-preference period and decreasing (8/9) in the post-preference period. Although the effects of alcohol stimulation on the fMRI signals were complex (blood flow, blood volume, anesthetic effects of alcohol, etc.), we can see from the changes in the trend of the periods that the effects varied with the process of preference formation.

### 3.4. Different Patterns of Functional Connectivity Related to Various Alcohol Conditions

The ANOVA of the correlation coefficient revealed that there were significant effects of alcohol training on the connections between several brain regions. The whole comparison results are provided in Table S3. Among the different changes, there were three different variation patterns for functional connectivity. First, three pair regions (NAc-IC, OT-Hypo, DB-VLPO) showed significant increases in the P-Post period before the alcohol injection (RS) compared to the P-Pre period (NAc-IC: Pre- vs. Post-, *p* < 0.001, OT-Hypo: Pre- vs. Post-, *p* < 0.05, DB-VLPO: Pre- vs. Post-, *p* < 0.05) but decreased after alcohol injection stimulation (IS) (NAc-IC: Pre- vs. Post-, *p* < 0.05, OT-Hypo: Pre- vs. Post-, *p* < 0.05, DB-VLPO: Pre- vs. Post-, *p* < 0.05) (Figure 6B). Second, there was no significant difference between the P-Pre and P-Post periods. However, the correlation coefficients were significantly different from the P-Mid and P-Pre/P-Post periods. The connection between the SNC (compacta of substantia nigra)-Tha (RS) and SNC-VTA (IS) was only observed to decrease and increase, respectively, in the P-Mid period (*p* < 0.05) (Figure 6C). Thus, the region of the SNC should be related to the formation of alcohol preference. Lastly, changes of the functional connectivity were only observed after alcohol stimulation but were not observed in the resting state. The coefficient of the NAc-Hypo (IS) in the P-Post state increased significantly (*p* < 0.05) compared with the P-Pre state, while the coherence of the Au-Hipp (IS) decreased significantly from P-Pre/P-Mid to P-Post (*p* < 0.01~0.05) (Figure 6D). 

### 3.5. Functional Connectivity Related to Different Alcohol Preferences

**Figure 7:**
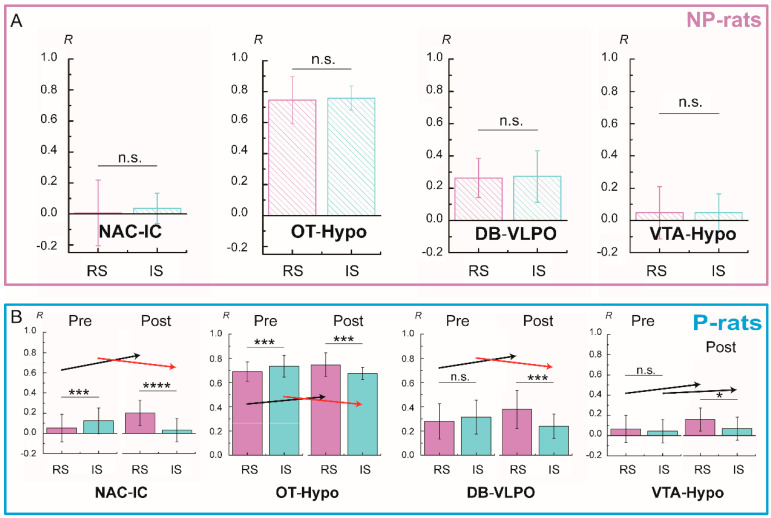
Comparison of the functional connectivity between the NP-rats (**A**) and P-rats (**B**). The black arrow represents an upward trend, while the red one is a downward trend. The abbreviations of brain areas are the same as in the main text: NAc (nucleus of accumbens), OT (olfactory tubercle), VTA (ventral tegmental area), IC (insular cortex), Hypo (hypothalamus), DB (diagonal band), VLPO (ventrolateral preoptic nucleus); * *p* < 0.05, *** *p* < 0.005, **** *p* < 0.001.

Among the changes of the functional connectivity related to alcohol stimulation or alcohol preference periods, there was a significant difference between the alcohol-preferring rats and the alcohol-non-preferring rats. After the acute alcohol stimulation, the functional connectivity of the NAC-IC, OT-Hypo, DB-VLPO, and VTA-Hypo was observed in the alcohol-preferring rats, especially for the first three pairs (*p* < 0.001, Figure 7B). However, there was no difference among these pair regions in the alcohol-non-preferring rats (Figure 7A). In addition, the opposite changes occurred with acute alcohol stimulation in the connectivity of the NAC-IC and OT-Hypo between different alcohol preference periods.

## 4. Discussion

In this study, brain-wide rsfMRI was used to investigate the changes of the functional connectivity caused by different animal strains (P- and NP-rats) and the different periods during the development of alcohol preferences for P-rats. The results of the variation of the functional network might be related to the development of alcohol preference caused by persistent alcohol drinking or the related genetic background. To our knowledge, this is the first time that the dynamic changes of the whole-brain-wide functional connections during the development of alcohol preference (from the first alcohol drinking to alcohol preference or addiction) have been described. To summarize, the following regions were mainly involved: NAc, Tu, VTA, IC, Hypo, SNC, Au, DB, Hipp, and VLPO. These regions are highly related to the behaviors of reward, motivation, emotion, moods, and cognitive circuits, which could independently or interactively lead to addictive or preference behaviors.

### 4.1. Influence of Alcohol Stimulation on the Brain Functional Connectivity

It is well-known that ethanol could serve as an anesthetic agent [27] and was used as such in ancient times. In addition, ethanol may have some unknown effects on the BOLD signal of the fMRI. To avoid the possibility that the changes of the brain connectivity are caused by ethanol’s effects, the changes of brain connections were only investigated under different states of alcohol preferences. 

The variation of the brain connectivity was almost opposite during different alcohol preference periods, which means that these changes were mostly related to the state of alcohol preference and were not caused by the alcohol’s anesthetic or hemodynamic effects. During the formation periods of alcohol preference, the following regions were involved: NAc, OT, VTA, IC, Hypo, SNC, Au, DB, Hipp, VLPO, and THA. Among these changes, several different systems were included, such as addiction and reward association (NAc, IC, OT, VTA, Hypo) [28,29,30,31], mood regulation (Hypo, DB, IC, AU, Tha) [32,33,34], wake-sleep cycles (VLPO, DB, SNC, Hypo) [35], memory (Hipp, DB, IC) [36,37], and motor activity (SNC, DB, VLPO, Tha) [38,39,40,41].

### 4.2. Functional and Structural Connections of Brain Regions

Although the investigation of the formation of the BOLD signal remains unresolved and the fMRI method could not be used to calculate the structural connection among different brain regions, it was important to analyze the changes of the functional connectivity during different states of alcohol drinking and determine the key regions associated with alcohol preference or addiction.

First, it can be seen from the results that the functional connectivity was partly related with the structural connectivity, and the brain regions with high functional connectivity coefficients tended to be those with strong structural connectivity. For example, the connections of some typical and representative regions (from basal ganglia to neocortex) are investigated in this study. The seventh region, the piriform cortex, had high correlations with the amygdala, insular cortex, and entorhinal cortex (the 11th, 13th, and 19th regions, respectively). These regions collectively performed in many olfactory-related behaviors or neurodegenerative diseases and had direct projections with each other [42,43]. Furthermore, the connection between the regions VTA (region 16) and SN (region 14) consisted of strong interactions, as these two brain regions were rich in dopaminergic neurons and contributed to reward circuits or reward-seeking behavior (for the SN, the main region was the SNc) [44].

In addition to the direct nerve connections, there were also intensive functional connections (BOLD–signal–dependence coherence) arising in parts of some regions, which did not have directed structural input or output connections [45]. These functional connections indicate that there were some complex factors involved in the BOLD signal analysis, such as mini-circuits, neurotransmitters, and multiple neurotransmitter-specific neuroplasticity circuits. Furthermore, the BOLD signal was used to indirectly measure the neural activity and is affected by multiple factors, including the local neural activity, metabolic capacity, the blood vessel system (blood flow and volume), and the neuroanatomy of the examined regions. Therefore, the BOLD signal may vary from the signals of neural activity measured directly by means of other technologies. 

### 4.3. Functional Connectivity and Alcohol Preference

In this study, the functional connectivity of brain regions NAc-IC, OT-Hypo, DB-VLPO, Au-Hipp, SNC-Tha, and SNC-VTA were highly relevant to alcohol preference during the alcohol training. Most of these regions have been mentioned in human alcoholics or animal models of alcohol dependence studies. For instance, NAc and its downstream ventral striatum, which is denoted as Hypo in this research, have been examined in studies of preference and drug abuse (dopamine, alcohol reward, or addiction) [46]. The thickness of the IC is altered in alcohol [47] and drug users [48]. DB is a brain region where acute or chronic ethanol administration increases the GABA-mediated inhibition of spontaneously active neurons (GABA receptor, alcohol-awake or asleep) [49]. VLPO, which mainly refers to sleep and motor activity, could be influenced by perinatal alcohol exposure (GABA receptor, alcohol-awake or asleep) [50]. 

Between the Pre-/Mid- and Post- periods, there is an opposite tendency with the RS and IS for the changes of the connectivity of NAc-IC, OT-Hypo, and DB-VLPO. In addition, the changes only occur during the state of stable preference or habituation (Post-state compared with the Pre-/Mid-state). This indicates that the synaptic plasticity of these regions, which is likely associated with alcohol expectation, changes during training (chronic alcohol use), and the craving would be fulfilled after alcohol drinking or injection. The changes of the connectivity coefficients from the resting state to the intoxicated state can be related to the changes in preference from a function level. Thus, the counter-connective trend of NAc-IC, OT-Hypo, and DB-VLPO in RS (P-Pre and P-Post) and IS (P-Pre and P-Post) suggests that alcohol induces changes in the neural circuits (stable rises or falls), as well as itself into the circuits (reverse connection mode). This is similar to the homeostasis neural circuit or the different meaning of alcohol at different periods. The coefficient decrease of Au-Hipp in the IS and Post- period characterizes the effect on the senses and memory of alcoholic intoxication or chronic alcohol use, as the auditory cortex and hippocampus are critical for episodic verbal and spatial memory, as well as cognition [51]. Furthermore, the transient action of alcohol itself on the brain as a depressant, analeptic, and tolerance should also be considered.

Among these significant different connections, it is also worthwhile noting that the changes to SNC-Tha and SNC-VTA (common reward and addiction-related brain regions) only happened in the Mid- term. There are direct neural connections that are relevant to the dopaminergic system between these two pairs [52]. Therefore, the changes might suggest that the pathway of the dopamine system among these regions may only take effect during the period when addiction is developing rather than when the addiction is being maintained.

### 4.4. Comparison of the Genetic and Alcohol Drinking Factors on the Alcohol Preference

It is well-known that alcoholism can be affected by social, psychological, and genetic factors. Previous research has indicated that the heritability of liability for alcohol addictions is estimated to be at 51–59% [53], and is mainly caused by familial resemblance. The inbred rat strains of P- and NP-rats were the best models to study the variations of functional networks on alcohol preference related to family history and alcohol training. Both the P- and NP-rats originated from a randomly bred Wistar colony [54]. These two kinds of animals have been used to study alcohol preference-related behaviors for many years [55]. According to the results of the correlation of the functional connectivity in different groups, there were significantly greater differences between different strains (P-rats vs. NP-rat) (*p* < 0.05) regardless of alcohol drinking training or alcohol stimulation. This provides possible evidence to support the fact that genetic factors play an important role in alcohol addiction or preference. 

The difference in the brain connections could provide another important clue in investigating the underlying neurobiological mechanisms. Thus, the difference (Δr > 0.15 and r > 0.20) of functional connectivity among the P-rats before/after alcohol preference periods and the NP-rats were collected to study the molecular mechanism of alcohol addiction among difference strains (Table S2). Finally, the formation of alcohol addiction involves neuroplasticity and neuroadaptation with the molecular mechanism. To completely understand and cure this disease, the identification of the molecular and neurochemical relationships with the changes of connectivity of these relevant brain regions should be further investigated.

### 4.5. Limitations and Perspective

In this study, the changes of brain networks during the formation of alcohol preference were explored without focusing on gender differences and the influence of other psychiatric problems (such as stress). The cerebral functions for male and female animals were different, and we also found that the cerebral regional metabolic information for different genders of alcohol-preferring or alcohol-non-preferring rats was different [56]. However, only male animals were utilized in this study. This is the major limitation of this study. To explore the alcohol preferences of female animals, more experiments on female alcohol preferences should be studied in the future. 

During the alcohol preference training, the alcohol drinking ratio for a single animal was monitored every day. Thus, the single-housed method was always utilized to screen the alcohol-preferring animals [57,58]. However, single-cage feeding is the common approach to model stress. We cannot strictly exclude the influence of external factors caused by single-cage feeding on the brain networks. Thus, all the animals in this study were housed using the same method. 

## 5. Conclusions

To summarize, P- and NP-rats were used to analyze the variations of the whole-brain functional network during alcohol training with the fMRI technology. Here, the regions of the OT, NAC, IC, DB, VLPO, Hypo, VTA, and SNC were found to change at different stages of alcohol preference formation. Unlike previous clinical studies that focused on comparing alcohol addiction patients with healthy groups, this animal study focused more on the process of alcohol preference formation. Alcohol preference is highly correlated with many diseases, such as alcohol addiction. The brain regions we found hold promise as new research and therapeutic targets for alcohol abuse-related diseases. Furthermore, this study of P- and NP-rats of different family backgrounds also provides evidence that genetic factors are more influential in the development of alcohol preference than constant alcohol exposure. This evidence suggests that people with a genetic history of diseases such as alcohol addiction should be more careful about avoiding exposure to addictive substances. In conclusion, this study provides important implications for both the clinical prevention and early intervention of alcohol preference and addiction for the future clinical study of alcoholism therapy.

## Figures and Tables

**Table 1:** The correlation coefficients of the whole-brain functional connectivity between different rat strains (alcohol preferring and alcohol non-preferring) and conditions (0-week, 2-week, and 4-week alcohol drinking training).

	RS, P-Pre	IS, P-Pre	RS, P-Mid	IS, P-Mid	RS, P-Post	IS, P-Post	RS, NP	IS, NP
RS, P-Pre	1	0.96	0.940	0.934	0.920	0.919	0.856	0.860
IS, P-Pre	0.960	1	0.942	0.942	0.924	0.919	0.863	0.878
RS, P-Mid	0.940	0.942	1	0.931	0.936	0.920	0.862	0.863
IS, P-Mid	0.934	0.942	0.931	1	0.913	0.910	0.850	0.870
RS, P-Post	0.920	0.924	0.936	0.913	1	0.925	0.841	0.848
IS, P-Post	0.919	0.919	0.920	0.910	0.925	1	0.857	0.869
RS, NP	0.856	0.863	0.862	0.850	0.841	0.857	1	0.908
IS, NP	0.860	0.878	0.863	0.870	0.848	0.869	0.908	1

Note: Alcohol-preferring rats: RS, P-Pre: resting state before alcohol preference; IS, P-Pre: acute alcohol stimulation before alcohol preference; RS, P-Mid: resting state after 2-week alcohol training; IS, P-Mid: acute alcohol stimulation after 2-week alcohol training; RS, P-Post: resting state after 4-week alcohol training; IS, P-Post: acute alcohol stimulation after 4-week alcohol training. Alcohol-non-preferring rats: RS, NP: resting state; IS, NP: acute alcohol stimulation. Same background: correlation coefficients in the same rat strains under different conditions (alcohol preference periods or acute alcohol stimulation).

## Data Availability

The datasets generated to obtain the results presented in this article are available from the corresponding authors on reasonable request (jie.wang@wipm.ac.cn/fuqiang.xu@wipm.ac.cn).

## Supplementary Materials

The following are available online at https://www.mdpi.com/article/10.3390/brainsci11111474/s1, Table S1: Collection of significant changes of the functional connectivity after ethanol stimulation comparing with the rest state during the periods of Pre-0 and Post-4 weeks alcohol training; Table S2: Collection of the difference of functional connectivity among P-rats before/after alcohol preference periods and NP-rats (Δr > 0.15 and r > 0.20). Table S3: Results of the statistical analysis of the functional connectivity in during the different periods of alcohol training (File name: ‘rs.xlsx’) and acute alcohol administration (File name: ‘is.xlsx’).
