# Supplementary material for: Variations of Brain Functional Connectivity in Alcohol-Preferring and Non-Preferring Rats with Consecutive Alcohol Training or Acute Alcohol Administration
Source: Brain Sci. 2021 Nov 7;11(11):1474. doi: 10.3390/brainsci11111474 (PMC8615902; doi:10.3390/brainsci11111474)
Supplement: Supplementary file 1 [file brainsci-11-01474-s001.zip › brainsci-1373881-supplementary/Supplemental materials2021.pdf]

Table S1: Collection of significant changes of the functional connectivity after ethanol stimulation comparing with the rest state during the periods of Pre-0 and Post-4 weeks alcohol training.

Training Days(P-Pre)				Training Days (P-Post)			
Brain region number	Brain region number	RS(*r*)	IS(*r*)	Brain region number	Brain region number	RS(*r*)	IS(*r*)
2	13	0.0532	0.1245 ↑	2	13	0.2012	0.0329 ↓
4	23	0.0287	0.1222 ↑	3	4	0.1003	-0.0414 ↓
4	26	0.6878	0.7338 ↑	3	8	0.1333	0.2635
8	22	0.1366	0.0191	3	27	0.1358	0.0427 ↓
9	22	0.1455	0.2131 ↑	4	26	0.7453	0.6738 ↓
17	19	0.2779	0.3416 ↑	8	24	0.3449	0.2506 ↓
20	22	0.3212	0.2235	9	20	0.2889	0.1744 ↓
21	28	0.072	0.1970 ↑	10	15	0.3775	0.2399 ↓
21	22	0.2155	0.1210	16	26	0.1581	0.0697 ↓
22	23	0.1888	0.2705 ↑				
26	27	0.4265	0.4843 ↑				

Note: r: correlation coefficient. RS: resting state; IS: intoxicating state.

Brain region number: 2. Accumbens Nucleus; 3. Caudate Putamen; 4. Olfactory Tubercle; 8. Somatosensory Cortex; 9. Auditory Cortex; 10. Diagonal Band; 13. Insular Cortex; 15. Preoptic Nucleus; 16. Ventral Tegmental Area; 17. Perirhinal Cortex; 19. Entorhinal Cortex; 20. Temporal Associatin Cortex; 21. Superior and Inferior Colliculus; 22. Visual Cortex; 23. Retrosplenial Cortex; 24. Motor Cortex; 26. Hypothalamus; 27. Thalamus; 28. Hippocampus.

Table S2: Collection of the difference of functional connectivity among P-rats before / after alcohol preference periods and NP-rats (Δr > 0.15 and r > 0.20).

Training period (P-Pre)				Training period (P-Post)			
Brain region number	Brain region number	RS(*r*) P-rats	RS(*r*) NP-rats	Brain region number	Brain region number	RS(*r*) P-rats	RS(*r*) NP-rats
6	23	0.2406	0.0796	2	13	0.2013	0.0364
9	22	0.1455	0.3274	3	24	0.2125	-0.0044
20	21	0.0445	0.2391	5	23	-0.2326	-0.0582
21	22	0.2155	0.0175	7	13	0.4314	0.1312
				9	22	0.1427	0.3274
				14	27	0.2804	0.1148
				23	24	0.2757	0.0742

Note: r: Correlation coefficient, RS: resting state. Brain region number: 2. Accumbens Nucleus; 3. Caudate Putamen; 5. Prelimbic Cortex; 6. Cingulate Cortex; 7. Piriform Cortex; 9. Auditory Cortex; 13. Insular Cortex; 14. Substantia Nigra; 20. Temporal Associatin Cortex; 21. Superior and Inferior Colliculus; 22. Visual Cortex; 23. Retrosplenial Cortex; 24. Motor Cortex; 27. Thalamus.

Table S3: Results of the statistical analysis of the functional connectivity in during the different periods of alcohol training (File name: ‘rs.xlsx’) and acute alcohol administration (File name: ‘is.xlsx’). *Note: As the data is too long, and it was uploaded as two excel files.*
